# Oral Immunotherapy Reshapes Intestinal Immunosuppression via Metabolic Reprogramming to Enhance Systemic Anti‐Tumor Immunity

**DOI:** 10.1002/advs.202302910

**Published:** 2023-10-26

**Authors:** Xinran Cao, Yuan Xu, Chen Zhou, Jiawei Huo, Shenge Su, Lei Liu, Ziran Zhu, Lei Li, Wang Jia, Chunru Wang, Mingming Zhen

**Affiliations:** ^1^ Beijing National Laboratory for Molecular Sciences Key Laboratory of Molecular Nanostructure and Nanotechnology Institute of Chemistry Chinese Academy of Sciences Beijing 100190 China; ^2^ University of Chinese Academy of Sciences Beijing 100049 China; ^3^ Beijing National Laboratory for Molecular Sciences Laboratory of Polymer Physics and Chemistry Institute of Chemistry Chinese Academy of Sciences Beijing 100190 China

**Keywords:** cellular metabolism, immune‐enhancing fullerenes, intestinal immunosuppression, proteomics, tumor immunotherapy

## Abstract

Tumor immunotherapy offers a new paradigm to treat cancer; however, the existing regimens are accompanied by the dilemma of insufficient therapeutic outcomes and off‐target adverse effects. The intestinal immune system contains a bulk of immune cells, which can be important contributors to the maintenance of systemic immune homeostasis. However, manipulating intestinal immunity to achieve systemic anti‐tumor immunity is extremely challenging. Here, an oral immunotherapy strategy is reported using immune‐enhancing fullerenes (IEF) that can reinvigorate anti‐tumor immunity via immune cell‐metabolic reprogramming of intestinal immune cells. Findings show that IEF can remodel anti‐inflammatory macrophages into tumor‐killing macrophages by regulating the energy metabolism pathway from oxidative phosphorylation (OXPHOS) to glycolysis. Consequently, IEF can reprogram the immunosuppressive intestinal immunity and enhance sys temic immunity in vivo, thereby boosting anti‐tumor immunity and converting “cold” tumors into “hot” tumors. Oral immunotherapy strategy, modulating autoimmune cells in the intestine and achieving systemic anti‐tumor immunity, can ensure safe and efficient tumor immunotherapy.

## Graphical abstract

In order to address the immunosuppressive microenvironment in the ileum of tumor‐bearing mice to enhance systemic immunity (a), immune‐enhancing fullerenes (IEF) is designed. After oral administration of IEF, it acts on the ileum and activates immune cells by affecting energy metabolism to boost the systemic immunity of the body and realize effective tumor killing (b).

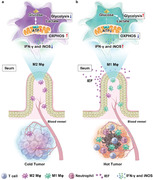

## 1 Introduction

Tumor immunotherapy has been widely used in clinical trials by enhancing the body's intrinsic immune system for killing tumors.^[^ ^1^ ^]^ Currently, the representative therapies mainly include adoptive cell therapy (ACT) and immune checkpoint blockade (ICB), which greatly improve the survival rate of most patients with cancer. ACT provides autologous or allogeneic T cells, edited and expanded in vitro, to patients with cancer.^[^ ^2^, ^3^, ^4^, ^5^ ^]^ However, it has limited therapeutic effects in solid tumors and its operation steps are tedious.^[^ ^4^, ^5^, ^6^ ^]^ On the other hand, ICB blocks the immunosuppressive pathway of T cells or tumor cells and enhances the systemic anti‐tumor immune response.^[^ ^5^, ^7^ ^]^ However, it is less effective in patients with immunosuppressive “cold” tumors, which have low levels of antigen‐specific T cells at the tumor site.^[^ ^8^ ^]^ Both ACT and ICB are commonly associated with serious immune‐related adverse events. Thus, novel tumor immunotherapy strategies for enhancing tumor immune response with high efficiency and safety need to be explored urgently.

The mucosal immune system is the largest immune tissue in the body. The intestinal mucosa contains a large number of lymphocytes (70% of the total lymphocytes in the body) and intrinsic immune cells, playing a crucial role in systemic immune homeostasis.^[^ ^9^ ^]^ Particularly, the immune cells in the gut have contact with the whole body through the peripheral blood system or lymphatic system.^[^ ^10^ ^]^ This offers the possibility to modulate immune cells at the intestinal site, thereby achieving systemic immune regulation. Normally, intestinal tissues secrete a bulk of anti‐inflammatory factors in order to resist the complex microenvironment of the gastrointestinal tract (commensal microorganisms, food, pathogens, etc.), resulting in the formation of an immunosuppressive microenvironment in the intestine.^[^ ^10^, ^11^ ^]^ Hence, activating immune cells in the intestine and reshaping the intestinal immunosuppressive microenvironment might enhance the body's immunity and provide a new strategy for tumor immunotherapy.

Accumulating studies show that the specific energy metabolism of immune cells controls their growth, differentiation, and activation.^[^ ^12^, ^13^, ^14^ ^]^ In particular, tumor‐killing immune cells, including M1‐type macrophages, neutrophils, and cytotoxic T cells, mainly rely on a glycolytic metabolism to rapidly generate energy. While anti‐inflammatory M2‐type macrophages and resting dendritic cells (DC) follow an oxidative phosphorylation (OXPHOS) metabolism pattern,^[^ ^12^, ^14^, ^15^, ^16^ ^]^ polarization of M2 to M1 macrophages can be facilitated by regulating the metabolic dynamics of M2‐type macrophages. Overall, we can regulate cellular metabolic dynamics to activate immune cells in the intestine and remodel the intestinal immunosuppressive microenvironment.

**Figure 1:**
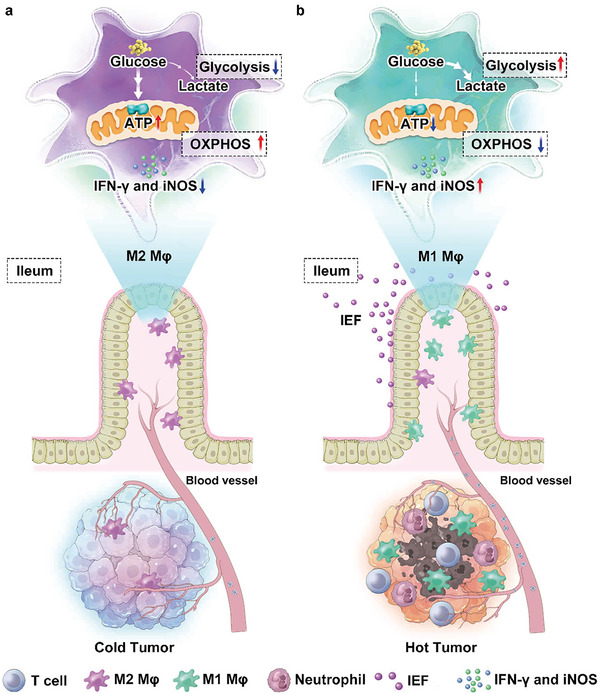
IEF reshape intestinal immunosuppression via metabolic reprogramming to enhance systemic anti‐tumor immunity. a) Immunosuppressive microenvironment in intestine and tumor. b) IEF activate immunity via immune‐metabolic reprogramming.

To achieve these goals, we explored oral immunotherapy to reshape the intestinal immunosuppressive microenvironment and enhance systemic anti‐tumor immunity via metabolic reprogramming (**Figure** **1**). We used fullerene, which can activate immune cells,^[^ ^17^ ^]^ as the key ingredient, mixed with some pharmaceutical excipients to design immunoenhancement fullerenes (IEF) for oral administration. For the first time, we demonstrated that IEF could convert tumor‐promoting immune cells with OXPHOS metabolism into anti‐tumor type immune cells with glycolytic metabolism by diminishing OXPHOS. In zebrafish and mouse xenograft tumors, IEF exhibited notable anti‐tumor and anti‐metastasis effects. In addition, IEF could act directly on the gut, rebuild the immunosuppressive microenvironment in the intestine, and enhance the systemic immune function of the body. Subsequently, the robust immune response in the tumor was greatly enhanced, achieving excellent tumor‐killing effects without significant immune‐related side effects. The oral immunopotentiator was validated to have a high potential for clinical translation in tumor immunotherapy by modulating its own immune cells to achieve safe and efficient anti‐tumor effects.

## 2 Results and Discussion

### 2.1 Formulation, Characterization, and Macrophage Polarization Function of IEF

**Figure 2:**
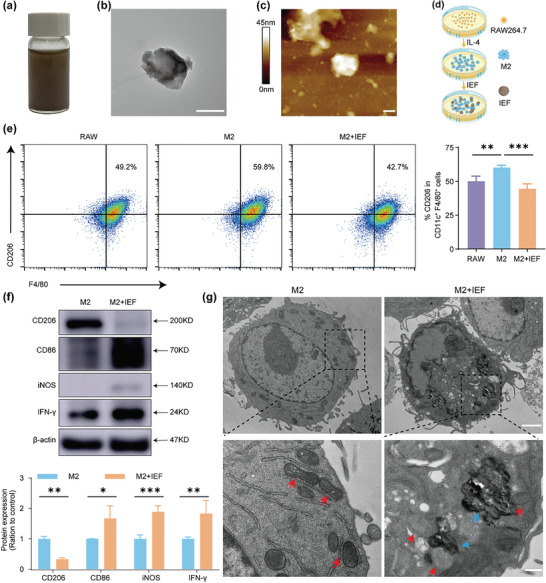
Synthesis, characterization of IEF and macrophage polarization in vitro. a) Photographs of IEF in water. b) TEM image of IEF. Scale bar, 500 nm. c) AFM image of IEF. Scale bar, 400 nm. d) Schematic diagram of co‐incubation of IEF with M2‐type macrophages. e) Representative flow cytometric analysis of CD206 in CD11c^+^ F4/80^+^ cells and corresponding quantification results in different group (*n* = 4, RAW group is RAW 264.7 cells). f) The protein expressions of M2 marker (CD206), and M1 markers (CD86, iNOS, and IFN‐γ) in M2‐type macrophages before and after co‐incubation with IEF (*n* = 3). g) The TEM image of M2 macrophages and the M2 macrophages after co‐incubation with IEF (red arrows, mitochondria. blue arrows, IEF). Scale bar, 2 µm (top) and 400 nm (bottom). The data are shown as the mean ± s.d. Student's t‐test, ^*^ *p* < 0.05, ^**^ *p* < 0.01, ^***^ *p* < 0.001.

IEF was prepared by mixing *C*_60_ fullerene powders, silicon dioxide (SiO_2_), co‐polyvidone (PVP/VA), microcrystalline cellulose (MCC), and other pharmaceutical excipients, and pressed into tablets (Figure S1, Supporting Information). For ease of administration on cells and mice, IEF was dispersed in water to form a dispersion (**Figure** **2**a). We used transmission electron microscopy (TEM) and atomic force microscopy (AFM) to observe the morphology and size of IEF. IEF suspension was found to have irregular spheroids with a size of 1.5 ± 0.5 µm (Figure 2b,c). To investigate the cytotoxicity of IEF, we co‐incubated IEF with cells, such as IEC‐6, L02, and HUVEC for 24 h and assayed their cell viabilities using a cell counting kit‐8 (CCK‐8). IEF was demonstrated to not be significantly toxic to normal cells (Figure S2, Supporting Information).

Next, we investigated the effect of IEF on macrophage polarization in vitro. Flow cytometry results revealed that RAW264.7 cells (murine macrophage leukemia cells, RAW) showed a notable increase in M2‐type macrophages (F4/80^+^ CD206^+^) after interleukin‐4 (IL‐4) stimulation. Following co‐incubation of IEF, M2‐type macrophages were found to be significantly decreased (Figure 2d,e). Subsequently, we confirmed the influence of IEF on macrophages by western blot (WB). RAW cells were incubated with IEF, and protein expression of representative markers, including M2‐type macrophage marker (CD206) and M1‐type macrophage markers ^[^ ^18^ ^]^ (CD86 and inducible nitric oxide synthase (iNOS)), was detected. The results suggested that IEF significantly reduced the protein expression of CD206, and increased the protein expression of CD86 and iNOS (Figure S3, Supporting Information). In M2‐type macrophages, IEF could reduce the protein expression of M2‐type macrophage markers and enhance that of M1‐type macrophage markers (Figure 2f). The results collectively indicated that IEF could remodel both RAW 264.7 cells and M2‐type macrophages to M1‐type macrophage transformation. Observation of the morphology of M2 macrophages after co‐incubation with IEF, by transmission electron microscopy (TEM), revealed that IEF affected the morphology and number of mitochondria in M2 macrophages (Figure 2g). Besides that, IEF decreased the ratio of red fluorescence of JC‐1 (a mitochondrial membrane potential detection probe) to green fluorescence, suggesting that its incubation with IEF greatly reduced the mitochondrial membrane potential and affected the mitochondrial function of M2 macrophages (Figure S4, Supporting Information). Since mitochondria serve as the main site of cellular oxidative phosphorylation metabolism, reducing the number of mitochondria would reduce intracellular energy production and thus affect cellular function.^[^ ^12^, ^15^ ^]^

### 2.2 Proteomics Profile of M2‐Type Macrophages Treated with IEF

**Figure 3:**
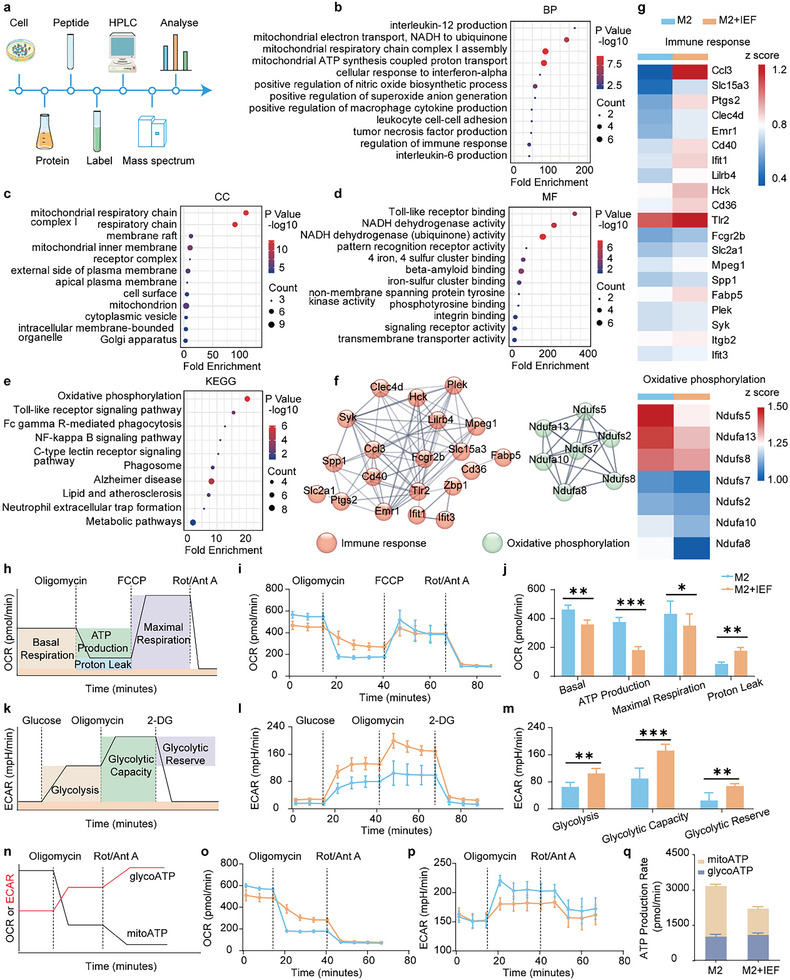
Analysis of quantitative proteomics and energy metabolism in M2‐type macrophages treated with IEF. a) The schematic diagram of proteomics in M2‐type macrophages treated with IEF. b–d) GO annotation of differential proteins for BP (b), CC (c), and MF (d). e) Enrichment of differential proteins on KEGG pathways. f) Interaction network analysis of differential proteins. g) Heat map of differential proteins expression. h) The schematic diagram of OCR assessment by a Seahorse XFe96 cell energy metabolism analyzer. i) OCR of M2‐type macrophages after IEF treatment. j) Corresponding calculated parameters of mitochondrial respiration in M2‐type macrophages after IEF treatment (*n* = 6). k) The schematic diagram of ECAR assessment. l) ECAR of M2‐type macrophages after IEF treatment. m) Corresponding calculated parameters of glycolytic capacity in M2 macrophages after IEF treatment (*n* = 6). n) The schematic diagram of ATP production rate assessment. o–q) The measurement flow and quantitative analysis of mitochondrial ATP (mitoATP) production rate and glycolytic ATP (glyATP) production rate of M2‐type macrophages after IEF treatment (*n* = 6). The data are shown as the mean ± s.d. Student's t‐test, ^*^ *p* < 0.05, ^**^ *p* < 0.01, ^***^ *p* < 0.001.

To further confirm the mechanism of macrophage polarization by IEF, we performed quantitative proteomics using the tandem mass tag (TMT) to detect the differential proteins between M2‐type macrophage (M2) and M2‐type macrophages treated with IEF (M2+IEF) groups (**Figure** **3**a). We found 153 differential proteins (fold change >1.2 or <0.8, *p*‐value < 0.05) by Gene Ontology (GO) enrichment analyses, including biological process (BP), cellular component (CC), and molecular function (MF). The results of BP analysis showed the differential proteins to be primarily enriched in interleukin‐12 production and cellular response to interferon‐alpha, which were involved in immune‐related pathways. The others were enriched in mitochondrial electron transport from Nicotinamide adenine dinucleotide (NADH) to ubiquinone, mitochondrial respiratory chain complex I assembly, and mitochondrial ATP synthesis coupled proton transport, which were associated with the mitochondrial oxidative phosphorylation pathway (Figure 3b). In addition, CC and MF analyses showed the differential proteins to be mainly concentrated in the mitochondrial respiratory chain and Toll‐like receptor binding and NADH dehydrogenase activity, respectively (Figure 3c,d). Further, we analyzed the differential proteins in the Kyoto Encyclopedia of Genes and Genomes (KEGG) pathway and found them to be predominantly gathered in oxidative phosphorylation, Toll‐like receptor signaling pathway, Fc gamma R‐mediated phagocytosis, and NF‐kappa B signaling pathway (Figure 3e). To further analyze the close relationship across the differential proteins and identify the core differential proteins, a protein–protein interaction network (PPI) was used. Twenty‐seven proteins were screened out with a high confidence of 0.7, and disconnected nodes were rounded off, being further divided into two clusters, including immune response and oxidative phosphorylation (Figure 3f). These differential proteins were then expressed separately in the form of heat maps (Figure 3g). The differential proteins assigned to immune response were all found to be increased in M2‐type macrophages after being co‐cultured with IEF. As for the oxidative phosphorylation pathway, IEF was found to reduce the expression of oxidative phosphorylation‐related proteins in M2‐type macrophages.

### 2.3 IEF Reprogrammed Macrophage Metabolism

Based on the proteomics profiles, we further monitored the cellular metabolism of mitochondrial oxidative phosphorylation in the M2‐type macrophages affected by IEF using a Seahorse XFe96 cell energy metabolism analyzer. Mitochondrial oxidative phosphorylation was assessed by oxygen consumption rate (OCR) after treatment with an ATP synthase inhibitor (oligomycin), H^+^ ionophore (the cyanide p‐trifluoromethoxyphenyl‐hydrazone [FCCP]), and electron‐transport chain inhibitor (rotenone and antimycin A, Rot/Ant A, Figure 3h). Results revealed that IEF treatment significantly diminished the basal respiratory, total ATP production, and maximal respiratory capacity of mitochondria in M2‐type macrophages accompanied by increased proton leak, which coincided with the above result of IEF incubation remarkably reducing the mitochondrial membrane potential (Figure 3i,j; Figure S4, Supporting Information). Furthermore, glycolysis capabilities, another type of metabolism in the cell, in M2‐type macrophages were evaluated and presented as the extracellular acidification rate (ECAR)^[^ ^19^ ^]^ (Figure 3k). Results showed that IEF notably enhanced the glycolysis, glycolytic capacity, and glycolytic reserve of M2‐type macrophages (Figure 3l,m). Additionally, the rates of ATP production by mitochondrial oxidative phosphorylation and glycolysis were detected (Figure 3n). IEF was found to primarily reduce the rate of ATP production by oxidative phosphorylation (mito‐ATP) while they barely affected the ATP production by glycolysis (glyco‐ATP) in M2‐type macrophages (Figure 3o–q) as well as in RAW cells (Figure S5, Supporting Information). The results indicated that IEF could affect macrophage mitochondrial function, diminish mitochondrial oxidative phosphorylation, and enhance glycolysis of M2‐type macrophages and RAW cells, resulting in the reprogramming of M2 and RAW intracellular metabolism, inducing their polarization toward M1‐type macrophages.

### 2.4 Potent Antitumor Efficacy of IEF both in Zebrafish and in Mouse Models

**Figure 4:**
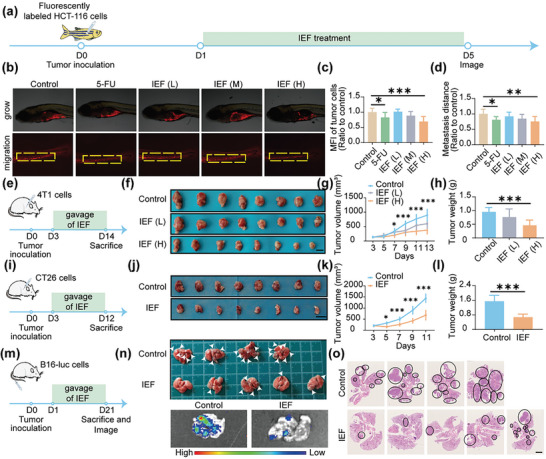
Anti‐tumor and anti‐tumor metastatic effects of IEF in zebrafish and mice. a) Scheme illustrating of anti‐tumor and anti‐tumor metastasis by IEF in zebrafish tumor xenografts models. b) Representative pictures of fluorescence images in PVS. c,d) Quantitative values of fluorescence intensity (c), and migration distance of HCT‐116 cells (d) (*n* = 8). e) Scheme of anti‐tumor process by IEF in subcutaneous 4T1 mouse tumor models. f) Photographs of tumors treated with different concentrations of IEF. Scale bar, 1 cm. g,h) Tumor growth curve (g), and tumor weights (h) of 4T1 tumor‐bearing mice (*n* = 8). i) Scheme of anti‐tumor process by IEF in subcutaneous CT26 mouse tumor models. j) Photographs of tumors treated with IEF. Scale bar, 2 cm. k,l) Tumor growth curve (k), and tumor weights (l) of CT26 tumor‐bearing mice (*n* = 8). m) Scheme of anti‐tumor metastasis process by IEF in melanoma lung metastasis mice models. n) Pictures and fluorescent imaging of mouse lung metastasis tumors obtained from day 21 (*n* = 5). o) H&E staining of lung tumors. Scale bar, 2.5 mm. The data are shown as the mean ± s.d. One‐way ANOVA, ^*^ *p* < 0.05, ^**^ *p* < 0.01, ^***^ *p* < 0.001.

Considering the superior immune activation effect of IEF, we investigated the anti‐tumor effects of IEF both in zebrafish and mouse models. For zebrafish tumor models, the fluorescent transfected human colon cancer cells HCT‐116 were transplanted into perivitelline space (PVS),^[^ ^20^ ^]^ and the anti‐tumor and anti‐metastatic effects of IEF were evaluated (**Figure** **4**a). Our results revealed that the fluorescence intensity in PVS after IEF and 5‐fluorouracil (a chemotherapeutic agent) treatments was significantly decreased than in the controls (Figure 4b,c). In addition, the distances migrated by tumor cells were calculated to evaluate the anti‐metastatic effect of IEF. The latter was found to significantly reduce the migration distance of tumor cells in zebrafish (Figure 4d). To investigate the anti‐tumor effects of IEF in mice, BALB/c mice bearing 4T1 murine breast tumors or CT26 colon tumors were established. When the tumor volume reached 100 mm^3^, mice were randomly grouped and administered IEF by gavage twice a day. We collected the tumor growth curves and assessed the anti‐tumor efficacy of IEF by measuring tumor weights at the end of the treatment. We observed that a high dose of IEF (50 mg kg^−1^) had inhibition rates of 51.2% and 61.0% in 4T1 and CT26 tumor‐bearing mice, respectively, without significant changes in weight (Figure 4e–l; Figure S6, Supporting Information). Hematoxylin and eosin (H&E) and Ki67 staining of tumor tissues further confirmed the significant tumor‐suppressive effect of IEF in CT26 tumor‐bearing mice (Figure S7, Supporting Information). Further, no palpable lesions were seen in the main organs, including the heart, liver, spleen, lung, and kidney, by H&E staining, which indicated that IEF caused no obvious harm to the body (Figure S8a, Supporting Information). Subsequently, we gave high doses of IEF to KM mice to observe the acute toxicity of IEF. Results showed no significant change in body weight and organ coefficient after IEF gavage, relative to that in the saline group. Based on these results, IEF was considered safe for administration (Figure S8b,c, Supporting Information). In addition, we established a melanoma lung metastasis model in mice by injecting B16‐Luc melanoma cells in mice via tail vein and evaluated the efficacy of IEF in inhibiting tumor metastasis (Figure 4m). At the end of the experiment, the lungs of tumor‐bearing mice were collected and imaged by in vivo fluorescence imaging. We observed that both the number and the fluorescence intensity of melanoma in the lung prominently declined after IEF treatment (Figure 4n). Moreover, the malignant melanoma cells were observably reduced by IEF (Figure 4o). The above results collectively indicated that IEF not only inhibited tumor growth and metastasis significantly but also had no significant side effects in mice.

### 2.5 IEF Remodeled the Intestinal Immunosuppressive Microenvironment

Before we explored the molecular mechanism of tumor treatment by IEF, we studied the biodistribution of IEF in mice using the liquid chromatography‐mass spectrometer (LC‐MS) to detect *C*_60_ contents in the main organs, blood, intestines, urine, and excrement. IEF was not detected in the liver, spleen, lung, kidney, blood, tumor, and urine, and was rather mainly distributed in the intestinal contents, and excreted out after 12 h (Table S1 and Figure S9, Supporting Information). This indicated that IEF could not be absorbed by the body Based on this, we speculated that IEF inhibits tumors by primarily remodeling the intestinal immunosuppressive microenvironment and activating the intestinal mucosal immune system. Thus, we systematically investigated intestinal immunity in multiple ways.

**Figure 5:**
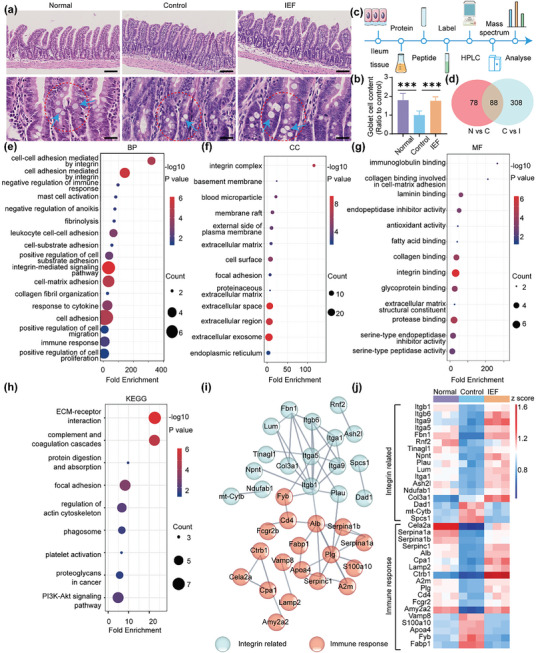
Proteomic assay in ileum. a) H&E staining of the ileum site. (Blue arrows, goblet cells). Scale bar, 100 µm (top) and 25 µm (bottom). b) The content of goblet cell in the ileum (*n* = 5). c) Flow chart of ileum proteomics. d) Venn analysis of differential proteins in three groups (N versus C represents Normal versus Control, C versus I represents Control versus IEF). e–g) Annotation of GO for BP (e), CC (f), and MF (g). h) KEGG analysis of differential proteins in ileum. i) PPI between differential proteins provided by STRING with high confidence in ileum. j) Heat map of differential proteins expressions in ileum (fold change >1.2 or <0.8, *p* < 0.01, *n* = 3). The data are shown as the mean ± s.d. One‐way ANOVA, ^*^ *p* < 0.05, ^**^ *p* < 0.01, ^***^ *p* < 0.001.

Considering that the ileum plays an important role in mucosal immunity,^[^ ^9^, ^21^ ^]^ we examined changes in the ileum before and after IEF treatment. H&E staining of the ileum of tumor‐bearing mice showed a significant reduction of goblet cells, which was significantly restored after IEF treatment (**Figure** **5**a,b). Goblet cells are mucus‐secreting cells distributed between mucosal columnar epithelial cells, whose main function is to synthesize and secrete mucin and form a mucosal barrier. A decrease in goblet cells in the ileum of tumor‐bearing mice resulted in decreased mucus secretion and an impaired mucosal layer barrier. The Impaired mucosal layer barrier results in impaired immunity of the intestinal mucosa, which occupies an important position in the immune system of the organism. After treatment with IEF, there was an increase in goblet cells in the ileum, which promoted mucus secretion and restored the integrity of the mucosal barrier. To further investigate the role of IEF in intestinal immune function, we used proteomics to detect changes in all proteins in the ileum after IEF treatment (Figure 5c). There were 88 differential proteins with a fold change >1.2 or < 0.8 and *p* < 0.01, overlapping in groups of normal versus control and control versus IEF treatment (Figure 5d). BP analysis revealed that the differential proteins mainly participated in cell adhesion mediated by integrin, integrin‐mediated signaling pathway, immune response, and positive regulation of cell migration (Figure 5e). Particularly, the differential proteins were significantly involved in integrin complex according to CC analysis (Figure 5f). In addition, the differential proteins were principally related to immunoglobulin binding, collagen binding, and integrin binding based on the MF analysis (Figure 5g). Subsequently, the KEGG analysis yielded that the above differential proteins were mainly enriched in complement and coagulation cascade, focal adhesion in the regulation of actin cytoskeleton (Figure 5h). The differential proteins were further imported into PPI and classified into two categories including integrin‐related pathway and immune response (Figure 5i). They are shown in a heat map, which revealed that most integrin‐related proteins as well as the immune‐related proteins were reduced in the ileum of tumor‐bearing mice than in the normal group and increased after treatment with IEF (Figure 5j). To further identify the effect of IEF on these two categories of proteins in ileum, we performed the following experiments, described below.

**Figure 6:**
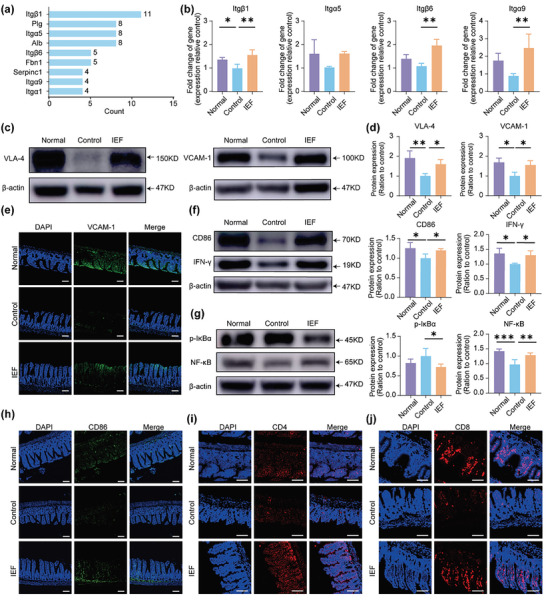
Measurements of ileum immunity and proteomic assay of serum. a) The count of connections per node protein related to integrin in PPI. b) The mRNA expressions of Itgb1, Itga5, Itgb6, and Itga9 in ileum (*n* = 3). c,d) The protein expressions and quantification of VLA4 and VCAM‐1 in ileum. e) Immunofluorescence staining of VCAM‐1 in ileum. Scale bar, 80 µm. f) The protein expressions and quantification of CD86 and IFN‐γ in ileum (*n* = 3). g) The protein expressions and quantification of p‐IκBα and NF‐κB in ileum (*n* = 3). h) Immunofluorescence staining of CD86 in ileum. Scale bar, 80 µm. i–j) Representative pictures of immunofluorescence staining for CD4^+^ T cells and CD8^+^ T cells staining in ileum. Scale bar, 160 µm. The data are shown as the mean ± s.d. One‐way ANOVA, ^*^ *p* < 0.05, ^**^ *p* < 0.01, ^***^ *p* < 0.001.

For integrin‐related pathway, we observed that the node counts of integrin β1 (Itgβ1), integrin α5 (Itgα5), integrin β6 (Itgβ6), and integrin α9 (Itgα9) were obviously high (**Figure** **6**a). Thus, we detected the gene expressions of these in the ileum by quantitative real‐time polymerase chain reaction (qRT‐PCR) and found that all of them were reduced in the control group while being notably enhanced after IEF treatment (Figure 6b). One of the important roles of integrin is to increase the adhesion between immune cells and vascular endothelial cells, thus promoting the recruitment of immune cells. Specifically, the very late activation antigen‐4 (VLA‐4, integrin α4β1) is a ligand for endothelial vascular cell adhesion molecule‐1 (VCAM‐1), and participates in the recruitment and migration of lymphocytes and monocytes.^[^ ^22^ ^]^ We examined the protein expression of VLA4 and its corresponding ligand VCAM‐1 in the ileum. Both were decreased in tumor‐bearing mice and increased after IEF treatments (Figure 6c,d). The immunofluorescence staining results of VCAM‐1 were consistent with the above conclusion (Figure 6e).

For immune response, we primarily detected the immune state of ileum using macrophages as one type of representative immune cells. Western blot results showed that the protein expression of M1‐type macrophage markers (CD86 and IFN‐γ) were reduced in the ileum of tumor‐bearing mice, and was remarkably increased after IEF treatment (Figure 6f). Given that the nuclear transcription factor‐κB (NF‐κB)‐related pathway was closely associated with M1‐type macrophage polarization,^[^ ^23^ ^]^ we examined the protein expression of phosphorylated IκB kinase‐α (p‐IκBα, an inhibitor of NF‐κB) and NF‐κB. Results revealed that the protein expression of p‐IκBα was reduced while that of NF‐κB was enhanced by IEF treatment (Figure 6g; Figure S10a, Supporting Information). The level of interleukin 12 (IL‐12) that was secreted by M1‐type macrophages was also enhanced after IEF treatment (Figure S10b, Supporting Information). Immunofluorescence images of CD86 were totally consistent with the above results (Figure 6h). With the increase and activation of M1 macrophages in the ileum, the number of T cells were increased remarkably (Figure 6i,j). The above results suggested that the M1‐type macrophages in ileum were significantly activated and the intestinal intrinsic and adaptive immune states were notably improved by IEF. This indicated that IEF could promote the adhesion and aggregation of immune cells to the ileum, stimulate the activation of immune cells, and rebuild the immunosuppressive microenvironment in ileum.

### 2.6 IEF Treatment Boosted Systemic Immunity and Anti‐Tumor Immunity in “Cold” Tumor

**Figure 7:**
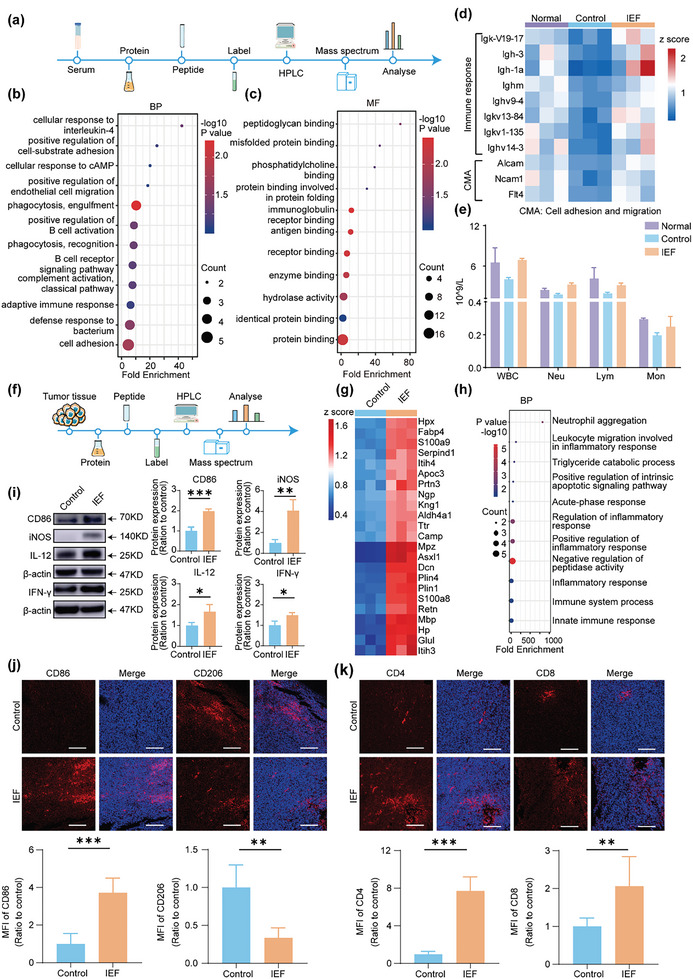
IEF treatment boost tumor immunity. a) Scheme of serum proteomics. b) Annotation of GO for BP in serum. c) Annotation of GO for MF in serum. d) Heat map of differential expression proteins in serum (fold change, >1.2 or <0.8; *p* < 0.01, *n* = 3). e) Routine blood tests for WBC, including neutrophils (Neu), lymphocytes (Lym), monocytes (Mon). f) Scheme of tumor proteomics process. g) Heat map of differential proteins in tumor tissues (fold change, >1.5 or <0.5; *p* < 0.01, *n* = 3). h) Annotation of GO for BP in tumor. i) WB assay and quantitative protein expressions of M1‐tpye macrophages related makers (iNOS, IL‐12, CD86 and IFN‐γ) in tumor (*n* = 3). j) Representative pictures and quantitative analysis of immunofluorescence staining for M1‐type macrophages (labeled by CD86) and M2‐tpye macrophages (labeled by CD206) in tumor (*n* = 5). MIF: mean immunofluorescence. Scale bar, 150 µm. k) Representative pictures and quantitative analysis of immunofluorescence staining for CD4^+^ T cells and CD8^+^ T cells staining in tumor (*n* = 5). Scale bar, 150 µm. Images were representative of four biologically independent mice in each group. The data are shown as the mean ± s.d. Student's t‐test, ^*^ *p* < 0.05, ^**^ *p* < 0.01, ^***^ *p* < 0.001.

Blood vessels are the key channels for the recruitment and migration of immune cells to effector sites. To observe the effects of IEF treatment on systematic immunity, we performed proteomics of serum (**Figure** **7**a). Results showed that the differential proteins of BP terms were enriched in cellular response to interleukin‐4, positive regulation of cell‐substrate adhesion, positive regulation of endothelial cell migration, phagocytosis, B cell activation, and so on (Figure 7b). For MF terms, they were enriched in immunoglobulin receptor binding, antigen binding, and so on (Figure 7c). Accordingly, the differential proteins were further classified into two categories. One was an immune response, including immunoglobulin heavy constant mu (Ighm), immunoglobulin heavy variable 9‐4 (Ighv9‐4), and immunoglobulin heavy variable V14‐3 (Ighv14‐3). The other category was cell adhesion and migration (CMA), including activated leukocyte cell adhesion molecule soluble isoform (Alcam), neural cell adhesion molecule 1 (Ncam1), and vascular endothelial growth factor receptor (Flt4). A significant increase in the two kinds of differential proteins was found in the IEF treatment group than in the control group (Figure 7d). Routine blood tests of mice showed that the number of white blood cells (WBC) of the tumor‐bearing mice was significantly reduced relative to that of normal mice, and the number of neutrophils, lymphocytes, and monocytes were also reduced. After IEF treatment, the WBC counts of the mice returned to the normal level, and the number of neutrophils, lymphocytes, and monocytes were also increased (Figure 7e). All the above results indicated that the systematic immunity was prominently boosted after IEF treatment.

To determine the effects on immune status in CT26 “cold” tumors after treatment with IEF, the proteomic profile of tumor tissues was evaluated (Figure 7f). The proteins with a fold change > 1.5 or < 0.5, and P < 0.01 were considered as differential proteins, and 197 differential proteins were screened out. Among them, 57 were primarily related to immunity, which were imported into PPI to observe the close relationship across the differential proteins. Twenty‐three proteins were obtained with a set of confidence score >0.7 and diffusion proteins were removed (Figure S11, Supporting Information). The differential proteins are shown in a heatmap (Figure 7g). We found that all 23 differential proteins were up‐regulated after IEF treatment. In addition, BP analysis of these differential proteins revealed that IEF treatment mainly affected neutrophil aggregation, positive regulation of inflammatory response, innate immune response, and so on (Figure 7h). Accordingly, we validated certain key differential proteins in tumors by WB. The neutrophilic granule protein (NGP), a negative regulator of tumor vascular development, mainly expressed in neutrophil precursors,^[^ ^24^ ^]^ was observably increased by IEF treatment. Additionally, expression of S100 calcium binding proteins A8 and A9 (S100A8 and S100A9), which were involved in neutrophil aggregation and the innate immune response,^[^ ^25^ ^]^ was significantly enhanced after IEF treatment. Further, the protein expression of cathelicidin antimicrobial peptide (Camp, which participates in the defense response to bacteria and in the innate immune response)^[^ ^26^ ^]^ was also boosted after treatment by IEF (Figure S12, Supporting Information). Subsequently, we determined the state of innate and specific immunity in tumor tissues. Our results revealed that the levels of M1‐type macrophage‐related markers, including CD86, iNOS, IL‐12, and IFN‐γ, were all increased after IEF treatment (Figure 7i). The counts of intrinsic immune cells, including neutrophils (labeled by LY6G) and M1‐type macrophages (labeled by CD86), were notably enhanced by IEF treatment, as seen by immunohistochemical or immunofluorescent staining (Figure S13, Supporting Information). In addition, the immunofluorescence results revealed that anti‐inflammatory M2‐type macrophages remarkably declined after IEF treatment. It was noteworthy that the levels of cytotoxic T cells (including CD4^+^ T cell and CD8^+^ T cell) were observably enhanced by IEF treatment (Figure 7j,k). The results collectively suggested that both innate and acquired immunity in tumor tissues were remarkably enhanced after IEF treatment.

## 3 Conclusion

In this study, we developed an oral immunotherapy primarily based on fullerenes, to enhance anti‐tumor immunity via immune‐metabolic reprogramming of intestinal immune cells. Specifically, our study revealed for the first time that IEF could inhibit intracellular OXPHOS and promote its conversion to a glycolytic metabolic mode, using macrophages as a representative. This induced the conversion of tumor‐promoting immune cells with OXPHOS as the main metabolic mode, such as M2, to tumor‐suppressive immune cells with glycolysis as the main metabolic mode, such as M1. Compared to the technically demanding and expensive disadvantages of ACT, the use of IEF in active immune cells is easy to operate, without causing severe cytokine release syndrome.

Nanomaterials have the potential to alter drug pharmacokinetics and are often used as carriers for loaded small molecule drugs,^[^ ^2^, ^6^ ^]^ which can enhance ICB therapy for oncology treatment. However, their potential as a platform for antitumor immunity still suffers from serious immune‐related side effects. The innovative aspect of the current work, was that oral immunotherapy could act directly on the gut to activate a large number of immune cells, enhancing systemic immunity and achieving superior tumor‐killing effects. Compared to other medicines given intravenously, oral administration has a higher adherence, can enhance intestinal and systemic immune responses,^[^ ^11^ ^]^ and has less side effects. Fullerene (as IEF) was found to be more stable to resist the breakdown by gastric acids and intestinal digestive enzymes, and could finally act on the ileum. It further activated the intestinal immune cells, reshaped the intestinal immunosuppressive microenvironment, and greatly enhanced systemic immunity. This promoted the adaptive immune response mediated by CD4^+^ T cells and CD8^+^ T cells in the “cold” tumor, enhancing the immunogenicity of the tumor site and converting it from a “cold” to “hot” tumor for superior tumor‐killing effect. Such an oral immunotherapy for achieving tumor‐killing effect by making full use of the body's own immune cells did not cause serious immune‐related side effects. On the other hand, compared to other nanomaterials, IEF showed great potential for clinical translation, owing to their simple production process, the absence of other by‐products, and established large‐scale manufacturing.

In conclusion, we presented a generalizable oral immunotherapy for tumor treatment. By modulating immune cell metabolism to achieve remodeling of the intestinal immunosuppressive microenvironment and enhancement of systemic immunity, IEF exhibited striking therapeutic benefits in a variety of tumor models. The oral immunotherapy achieved systemic immune enhancement by activating the body's own largest immune system, the mucosal immune system, avoiding complex in vitro manipulations and immune‐related side effects. This could pave the way for the development of a highly innovative and translational approach for tumor immunotherapy, and have implications in the treatment of other immunosuppression‐related diseases.

## 4 Experimental Section

### Synthesis and Characterization of IEF

IEF were synthesized as follows: C_60_, silicon dioxide (SiO_2_), co‐polyvidone (PVP/VA), microcrystalline cellulose (MCC), and other pharmaceutical excipients in the mass ratio of 20:50:7:3:20. SiO_2_ was an excellent flow promoter, which could increase the inter‐particle mobility during mixing for a more homogeneous mix. Co‐polyvidone represented were water‐soluble organic polymer compounds that were linear copolymers of N‐vinylpyrrolidone (NVP) and vinyl acetate (VA), and was used to increase the adhesion between the particles in the mixing process. MCC was an excellent emulsifier and disintegrating agents that could promote the rapid disintegration of IEF after oral administration, releasing the active ingredient C_60_ to play the corresponding role. The above materials were mixed well in a certain ratio and pressed into tablets by the ZP5A Rotary Tablet Press (Shanghai Tianhe Pharmaceutical Machinery, China) for easy storage and oral administration. In this experiment, IEF were dissolved in water in order to facilitate their administration into cells and mice. The morphology and size of IEF were characterized by TEM (Tecnai Spirit, FEI, USA) and AFM (MultiMode 8‐HR, Bruker, Germany).

### Cell Culture

Intestinal epithelial cells (IEC‐6), human liver normal cells (L02), human umbilical vein endothelial cells (HUVEC), RAW 264.7 macrophages, human colon cancer cells (HCT116), mouse breast cancer cells (4T1), mouse colon cancer cells (CT26), and mouse melanoma cells (B16‐luc) were purchased from the Institute of Basic Medical Sciences, Chinese Academy of Medical Sciences (China). IEC‐6, L02, and HUVEC were cultured in DMEM with 10% (v/v) FBS, 1% (v/v) penicillin (100 µg mL^−1^), and 1% (v/v) streptomycin (100 µg mL^−1^) in an incubator with 5% CO_2_ at 37 °C. RAW 264.7 macrophages were cultured in DMEM containing 10% FBS at 37 °C in an incubator containing 5% CO_2_. 4T1, CT26, and B16‐luc cells were cultured in RPMI‐1640 medium with 10% (v/v) FBS, 1% (v/v) penicillin (100 µg mL^−1^), and 1% (v/v) streptomycin (100 µg mL^−1^) in an incubator with 5% CO_2_ at 37 °C.

### Cytotoxicity Testing

IEC‐6, L02, or HUVEC cells were seeded in 96‐well plates at a density of 5 × 10^4^. After the cells were attached to the wall, different concentrations of IEF were added separately and incubated for 24 h. Finally, 100 µL of CCK solution was added to each well and the absorbance at 450 nm was measured after 1 h of incubation.

### Macrophage Culture and Polarization

For the culture of RAW macrophages, RAW 264.7 were cultured in large dishes, and after 24 h, the medium was replaced with fresh medium or medium containing IEF and co‐incubated for another 4 h. For the culture of M2 macrophages, RAW 264.7 were cultured in large dishes, and after 24 h, macrophages were stimulated with 20 ng mL^−1^ of IL‐4 for 24 h to polarize them into M2‐type macrophages. Subsequently, the medium was replaced with fresh medium or medium containing IEF (500 µg mL^−1^) and co‐incubated for 4 h.

### Flow Cytometry (FCM)

Approximately 1 × 10^6^ cells from RAW, M2, and M2+IEF groups were taken into a flow‐through tube. 2 mL PBS was added to wash the cells, which were then centrifuged at 500 g for 5 min. Monoclonal antibodies 0.25 ug CD11c (45‐0114‐82, Invitrogen, USA), 0.25 ug F4/80 (58‐4801‐82, Invitrogen, USA), and 0.125 ug CD86 (11‐0862‐82, Invitrogen, USA) were added to each tube, vortexed and mixed well, and incubated at 37 °C. Avoiding light for 15 min, 0.5 mL of fixation solution was added at the end of the incubation, kept at 37 °C, and avoiding light for another 20 min, was centrifuged at 500 g for 5 min. Two milliliters of 1× film‐breaking solution were added to resuspend the precipitate, which was then incubated in the dark at room temperature for 10 min, and centrifuged at 500 g for 5 min. One milliliter of 1× membrane‐breaking solution was added to resuspend the precipitate. Next, 0.125 ug of CD206 (MMR) antibody (12‐2061‐82, Invitrogen, USA) was added to each tube and incubated in the dark at room temperature for 30 min. One milliliter of 1× membrane‐breaking solution was added next, and after mixing by gentle blowing with buffer, the supernatant was centrifuged at 500 g for 5 min and discarded. In the end, 500 µL of PBS were added to re‐suspend the cells and then 30 000 cells were collected in each tube by flow cytometry.

### Western Blot (WB) Analysis

Detailed information of the antibodies used for WB is as follows: anti‐CD206 (24 595, Cell Signaling Technology (CST), USA), anti‐CD86 (19 589, CST, USA), anti‐iNOS (ab178945, Abcam, UK), anti‐IFN‐γ (ab133566, Abcam, UK), anti‐GAPDH (2118, CST, USA), anti‐β‐actin (4970, CST, USA), anti‐VLA4 (ab81280, Abcam, UK), anti‐VCAM‐1 (ab134047, Abcam, UK), anti‐IκB‐P (2859, CST, USA), anti‐NF‐κB (8242, CST, USA), anti‐IL‐12 (100 321, Sino Biological, China), anti‐NGP (600‐401‐GW9, Rockland, USA), anti‐CAMP (12009‐1‐AP, Proteintech, USA), anti‐S100A8 (ab92331, Abcam, UK), and anti‐S100A9 (26992‐1‐AP, Proteintech, USA).

RAW 264.7 cells were seeded in six‐well plates at a density of 1 × 10^5^, and after 24 h apposition, they differentiated into M2 cells; thereafter, co‐incubate the cells with IEF (500 µg mL^−1^) for 4 h. The medium was then discarded and the cells were collected. Lysis was performed in radioimmunoprecipitation assay lysis buffer (RIPA, P0013, Beyotime, China) containing protease phosphatase inhibitor mixture (P1048, Beyotime, China). For ileum and tumor tissues, an appropriate amount of tissue sample was weighed into a mortar (pre‐cooled with liquid nitrogen), added liquid nitrogen to fully grind it to powder, and then added RIPA lysis buffer to the extracted protein. Subsequently, the BCA protein quantification kit (P0010, Beyotime, China) was used to quantify the total protein.

Approximately 30 µg protein, in each well, was separated by 4–20% polyacrylamide gel electrophoresis gels submerged in running buffer, and then transferred from the gel to polyvinylidene fluoride (PVDF) membranes (0.45 µm, Millipore, USA) in transfer buffer. Subsequently, the membranes were blocked with 5% non‐fat milk for 1 h at room temperature. After rinsing with TBST, the membranes were incubated with the primary antibodies at 4 °C, with overnight shaking. Next day, the membranes were rinsed and incubated with the secondary antibodies at room temperature, shaking for 1 h, and then rinsed with TBST for imaging. Proteins were visualized by electrochemiluminescence (Millipore, USA) and imaging system (Tanon 4200, Tanon, China). Finally, the gray values of the bands were quantified.

### Transmission Electron Microscopy (TEM) of Cells

Cells were stored in 2.5% glutaraldehyde. They were subsequently soaked in 1% osmium tetroxide (2 h), dehydrated with ethanol fractionation (30, 50, 70, 80, 90, 100%, 100%, 7 min per stage), and again dehydrated with acetone (10 min, 2 twice). Cells was then allowed to be infiltrated by a fractional mixture of acetone and SPI‐PON812 resin (3:1, 1:1, 1:3), which was then replaced by pure resin. The sample was embedded in the resin with 1.5% BDMA. After 12 h polymerization at 45 °C and 48 h polymerization at 60 °C, the samples were sectioned with a micro‐tome (EM UC6, Leica, Germany). Finally, this study double‐stained the sections with uranyl acetate and lead citrate, and scanned each sample with TEM (Tecnai Spirit, FEI, USA).

### Proteomics Experiment and Bioinformatics Analysis

RAW 264.7 cells were seeded in six‐well plates at a density of 1 × 10^5^, and after 24 h apposition, they differentiated into M2 cells by different treatments, after which IEF (500 µg mL^−1^) was added for co‐incubation for 4 h. The medium was discarded thereafter and the cells were collected. For tissue samples, ileum and tumor were wiped clean and put in liquid nitrogen; the removed intestine was stripped the mesentery and cut longitudinally to clean the contents. Next, an appropriate amount of tissue sample was weighed into a mortar (pre‐cooled in liquid nitrogen) and liquid nitrogen was added to fully grind it to powder.

To the samples of each group, four times the volume of powdered lysis buffer was added, and lysed by ultrasound. Subsequently, the turbid liquid was centrifuged at 12 000 g for 10 min at 4 °C to remove the cell debris. Finally, the supernatant was collected and transferred to a new centrifuge tube, and the protein concentration of each sample was determined using the BCA kit (P0010, Beyotime, China).

Next, an equal amount of each sample protein was taken for enzymatic hydrolysis, added an appropriate amount of standard protein, and adjusted the volume to the same with lysis solution. TCA (L020000, Sigma–Aldrich, USA) at a final concentration of 20% was slowly added, vortexed to mix, and settled at 4 °C for 2 h. The turbid liquid was centrifuged at 4500 g for 5 min to discard the supernatant, and the precipitate was washed 2–3 times with pre‐cooled acetone. After drying the pellet, TEAB (140 023, Sigma–Aldrich, USA) was added at a final concentration of 200 mm, the pellet was dispersed ultrasonically, trypsin was added at a ratio of 1:50 (protease: protein, m/m), and the mixture was hydrolyzed overnight. Dithiothreitol (DTT) was added to a final concentration of 5 mm, and reduced at 56 °C for 30 min. Finally, iodoacetamide (IAA) was added to make the final concentration 11 mm, and incubated for 15 min in the dark at room temperature. For TMT label, the labeling reagent was dissolved in acetonitrile after thawing to mixed with the peptide, and incubated for 2 h at room temperature. When the labeled peptide was mixed, it was desalted and freeze‐dried in vacuum.

Next, peptide segments were graded according to the following steps: a step gradient of 8–32% acetonitrile, pH 9, was used, and the peptides were combined into six components for vacuum freeze‐drying. Subsequently, the peptides were dissolved in the mobile phase A of liquid chromatography and then separated using the EASY‐nLC 1200 ultra‐high performance liquid system, after which they were injected into the NSI ion source for ionization, and then taken for the Orbitrap Exploris 480 mass spectrometry analysis.

From mass spectrometry analysis, the mass‐to‐charge ratio and signal intensity of fragment ions were obtained after peptide fragmentation, and compared the results with the theoretical secondary spectrum database based on the protein sequence in UniProt. After completing the database search, a series of quality control evaluations were required to ensure that the quality of the results meets the standards, including peptide length distribution, peptide number distribution, protein coverage distribution, and protein molecular weight distribution. Based on the signal intensity value of each peptide in different samples from the database search, differential proteins were screened out between every two groups.

To identify whether the differentially expressed proteins had a significant enrichment trend in certain functional types, DAVID was used to get the GO annotation proteome and Gene Ontology enrichment analysis, including biological process, cellular component, and molecular function and KOBAS database to get the KEGG pathway enrichment analysis. The functional classification and pathways in which the differentially expressed proteins were significantly enriched (*p*‐value <0.05) were shown by means of bubble charts. Clustering analysis was based on the *p*‐value of the Fisher's exact test, obtained by enrichment analysis, using the hierarchical clustering method, to gather the related functions in different groups together and draw them as a heatmap. Finally, the differential proteins were imported into STRING, the online database platform for network interaction analysis.

### Seahorse Extracellular Flux Analyzer Assay

RAW 264.7 cells were incubated in Seahorse XFe96 cell culture microplates (Agilent, USA) at a density of 2 × 10^4^ for 24 h. Thereafter, the cells were cultured in a changed medium (RAW) or in a medium with IL‐4 to differentiate them into M2 cells. Subsequently, different reagents were added for different assays accordingly. The corresponding values were detected using the Seahorse XFe96 Analyzer (Agilent, USA).

For ECAR assay, 25 µL of glucose (G7528, Sigma, USA), 25 µL of 1.5 µmol L^−1^ oligomycin (ab141829, Abcam, USA), and 25 µL of 2‐deoxyglucose (2‐DG, D8375, Sigma, USA) were added sequentially. For OCR assay, 25 µL of 1.5 µmol L^−1^ oligomycin, 25 µL of 1 µmol L^−1^ mesoxalonitrile 4‐trifluoromethoxyphenylhydrazone (FCCP, C2920, Sigma, USA), and 25 µL of antimycin A (ab141904, Abcam, USA)/rotenone (R8875, Sigma, USA) were added sequentially. For real‐time ATP rate assay, cells were sequentially treated with 25 µL of 1.5 µmol L^−1^ oligomycin and 25 µL of antimycin A/rotenone.

### Mitochondrial Membrane Potential Assay

The mitochondrial membrane potential was detected using the JC‐1 staining kit (M8650, Solarbio, China) as follows: RAW 264.7 cells were seeded at a density of 1 × 10^4^ in a confocal dish and incubated for 24 h at 37 °C in an incubator. After the cells were walled, they were differentiated into M2 cell, and then IEF (500 µg mL^−1^) was added for 4 h. The culture medium was aspirated thereafter, and the cells were washed once with PBS, and then with 1 mL of culture medium. Next, 1 mL of JC‐1 staining working solution was added and mixed thoroughly, incubated for 20 min at 37 °C in a cell incubator, and the supernatant aspirated and washed twice with JC‐1 staining buffer. Thereafter, 1 mL of cell culture medium was added, and the fluorescence intensity at 488 nm versus that at 535 nm was observed by laser confocal microscopy.

### Zebrafish Transplantation Tumor Model

Zebrafish were purchased from Hangzhou Huante Biotechnology Co, China. Human colon cancer (HCT‐116) cells were labeled with red fluorescent dye and microinjected into the yolk sacs of 2 dpf wild‐type AB strains of zebrafish. Approximately 200 cells were transplanted per tail to establish a zebrafish human colon cancer transplantation model. The zebrafish injected with HCT‐116 were cultured at 35 °C until 3 dpf. At 3 dpf, zebrafish with good tumor cell consistency were selected under the microscope (SZX7, OLYMPUS, Japan). Next, they were randomly assigned to six‐well plates and divided into control group, 5‐fluorouracil (1000 µM) group, IEF low concentration group (156.25 µg mL^−1^), IEF medium concentration group (312.5 µg mL^−1^), and IEF high concentration group (625 µg mL^−1^). They were given the corresponding drug treatments for 2 days. Ten zebrafish were randomly selected from each group and photographed under a fluorescence microscope. The data were acquired using NIS‐Elements D 3.20 advanced image processing software to analyze the fluorescence intensity of tumor cells. The data were further acquired with Image J software to analyze the migration distance of tumor cells.

### Animal Models and Treatments

Female Balb/c mice (5 weeks old) and male KM mice (18‐22 g), were purchased from Beijing Huafukang Bioscience Co. Inc. (Beijing, China), and all mice were reared in a temperature‐controlled environment with a 12 h light‐dark cycle. All the experimental protocols involving live animals were reviewed and approved by the Animal Ethics Committee of the Institute of Chemistry, Chinese Academy of Sciences (approval number SYXK (Jing) 2018‐0033).

For the Mouse Model of Subcutaneous Tumor, the Operation was as Follows: 4T1 breast cancer cells and CT26 colon cancer cells were cultured to logarithmic growth phase, digested, and collected with trypsin, and washed and resuspended with normal saline to reach a final cell concentration of 2 × 10^7^ cells mL^−1^ suspension. Thereafter, 0.1 mL of cell suspension was injected into the mice subcutaneously under the axilla. Three days later, the mice were divided into four groups, namely a) normal group, b) control group + saline, c) IEF (20 mg kg^−1^), and d) IEF (40 mg kg^−1^) group. Saline and IEF were gavaged twice a day until the end of the experiment. Survival of the animals was observed daily, and body weight and tumor volume were measured every alternate day. After ≈12 days, the experiment ended, when the tumor volume reached 1000 mm^3^, calculated by the following formula: width ^2^ × length × 0.5.

For the acute toxicity of IEF, KM mice were randomly grouped according to body weight. The mice were divided into saline and IEF groups, with ten animals in each group. IEF was administered once, at a dose of 5000 mg kg^−1^ by gavage. Body weights of the mice were recorded on days 0, 1, 3, 7, and 14. On day 14, the experiment was terminated, the animals were executed, and major organs of the mice were removed and weighed to calculate the organ coefficient.

(1) OrganCoefficient=Organweight/Mousebodyweight

For the metastasis‐resistant mouse model, B16‐luc cells were cultured to logarithmic growth phase, digested and collected with trypsin, and then washed and resuspended in normal saline to reach a final cell concentration of 1 × 10^7^ cells mL^−1^ suspension. Thereafter, 0.1 mL of cell suspension was injected into mice via tail vein. The mice were randomly divided into two groups, namely a) control group + saline group and b) IEF (20 mg kg^−1^) group. Saline and IEF were gavaged twice a day, until the mice in the control group appeared dead and the experiment was finished.

### IVIS

The anti‐pulmonary metastasis assay was completed and sampling was started on day 20. At the time of sampling, 200 µL of 15 mg mL^−1^ D‐fluorescein sodium salt (D1007, US EVERBRIGHT INC, China) was injected intraperitoneally into the mice, and the lung tissue was removed after 10 min and imaged with a Xenogen IVIS Lumina in vivo imaging system (PerkinElmer, USA).

### Histopathology Examination

Tumor, intestine, and the main organs were fixed in formalin and embedded in paraffin wax blocks through a serial alcohol gradient. Next, 5 µm slices were cut and stained with H&E, with dewaxing, hydration, staining, dehydration, and sealing. The slide was then placed in the scanner (Nano Zoomer‐SQ, HAMAMATSU, Japan), and at least five areas were selected for per‐film for analysis.

### Immunohistochemistry (IHC) in Tumor

Antibodies for IHE were purchased from Abcam, including anti‐Ki67 (ab15580, Abcam, UK), and Cell Signaling Technology (CST), including anti‐LY6G (75 082, CST, USA) and anti‐CD86 (20 018, CST, USA).

Tumors were embedded in paraffin wax blocks. They were cut into 4 µm slices. After washing, antigen retrieval solution was used to repair tissue antigen, and 3% BSA (ST025, Solarbio, China) was used to block non‐specific protein binding sites. Primary antibody was used for specificity staining. After washing with PBS, they were incubated with secondary antibody, color rendered with DAB, and covered with coverslip.

### IEF Biodistribution Detected In Vivo

The heart, liver, spleen, lung, kidney, urine, intestine, contents of the intestine and blood were collected after the mice were given IEF by gavage at 1, 4, 8, and 12 h and urine and stool were collected within 12 h. Next, 2 mL of hydrochloric acid was added to an appropriate amount of each matrix sample, and the latter was digested at 75 °C for 30 min. O‐xylene solution was added after the sample was placed at room temperature, and it was vortexed for 10 min before liquid–liquid extraction. Next, the sample was centrifuged at 4000 rpm for 5 min at room temperature; 100 µL of the supernatant was mixed with 100 µL of acetonitrile, and the mixture was transferred to a sample bottle for injection, followed by the performance of LC‐MS/MS analysis.

Analyst 1.6.2 software was used to integrate, calculate, and process the chromatographic peaks. Taking the peak area of the analyte as the ordinate (y) and concentration as the abscissa (x), the weighted least squares method (weight coefficient of 1/x^2^) was used to perform regression calculation and calculate the concentration of the analyte.

### Immunofluorescence (IF) Assay

After the mouse ileum and tumors were removed, they were cleaned with saline, embedded with OCT, and cut into 4‐µm‐thick slices, which were left at room temperature and then washed with TBS. They were then soaked in PBST containing 5% skim milk powder to block the non‐specific binding sites. After washing with TBST, they were incubated with the corresponding primary anti‐VCAM‐1(ab134047, Abcam, UK), anti‐CD86(ab119857, Abcam, UK), anti‐CD4 (GB15064, Servicebio, China), anti‐CD8 (ab217344, Abcam, UK), and anti‐CD206(24 595, CST, USA) at 4 °C overnight, protected from light. This was followed by three washes with TBST and incubation with the corresponding secondary antibody. Finally, the slides were blocked with an anti‐fluorescence quencher containing 4,6‐diamidino‐2‐phenylindole, dihydrochloride (DAPI, for labeling nuclei) and covered with coverslip. The samples were then imaged with a laser confocal scanner. Average fluorescence was quantified using Image‐Pro Plus 6.0 (Media Cybernetics, USA).

### Blood Routine Test

Mouse orbital blood was collected, placed in an anticoagulant tube, and then used in an automated blood analyzer (BC‐5000VET, Mindray, China) to perform hematological analysis to detect white blood cell (WBC) indicators, mainly neutrophils, lymphocytes, and monocytes.

### ELISA

The proteins were extracted in PBS using protease inhibitors and phosphatase inhibitors. Protein concentration was quantified with BCA protein assay kit. Cytokines of NF‐κB and IL‐12 were detected in the ileum by ELISA kits (Neobioscience, China) according to the manufacturer's instructions.

### qRT‐PCR Assay

A certain amount of frozen ileal tissue was weighed, to which 1 mL of TRIzol reagent (Invitrogen, USA) was added; it was ground and crushed by a tissue grinder, shaken thoroughly, centrifuged at 12 000 g for 10 min at 4 °C, and the supernatant extracted. To the supernatant, 200 µL of chloroform (C/4920/17, Innochem, China) was added, shaken thoroughly, left to stand at room temperature for 5 min, and then centrifuged at 12 000 g for 15 min at 4 °C. Approximately 0.5 mL of pre‐chilled isopropanol (I1700, Innochem, China) was added to the supernatant, mixed thoroughly, precipitated for 10 min at room temperature, and centrifuged at 12 000 g for 15 min at 4 °C. After discarding the supernatant, 1 mL of pre‐chilled 75% DEPC ethanol was added, mixed thoroughly, centrifuged at 7500 g for 5 min at 4 °C, the supernatant discarded, and washed again with DEPC ethanol. The precipitate was dried at room temperature for 20 min; 20 µL of DEPC water was added according to the amount of precipitate, blown well, heated at 58 °C for 10 min, and then centrifuged instantaneously to obtain the total RNA.

The sample solution containing 1 µg of RNA was added to the PCR tube, after which 1 µL of gDNA was added and the volume made up to 10 µL with RNase‐free water; thereafter, 10 µL of first strand cDNA synthesis superMix (Novoprotein, China) was added, gently mixed and transiently centrifuged, and the RNA was reverse transcribed by programmed warming at 50 °C for 15 min and 75 °C for 5 min, to cDNA.

Finally, 10 µL of SYBR, 1.2 µL of mixed solution containing 10 µm upstream primer and downstream primer, 1 µL of cDNA sample solution, 0.4 µL of ROX II, and 7.4 µL of RNase‐free water were added to the octuple rows of tubes. Expression levels of the target protein mRNA were detected in a qPCR detection system (CFX96, BioRad, USA).

Itgb1 forward primer: 5′‐ ATGCCAAATCTTGCGGAGAAT‐3′;

Itgb1 reverse primer: 5′‐ TTTGCTGCGATTGGTGACATT‐3′;

Itga5 forward primer: 5′‐ CTTCTCCGTGGAGTTTTACCG‐3′,

Itga5 reverse primer: 5′‐ GCTGTCAAATTGAATGGTGGTG‐3′;

Itgb6 forward primer: 5′‐ CAACTATCGGCCAACTCATTGA‐3′,

Itgb6 reverse primer: 5′‐ GCAGTTCTTCATAAGCGGAGAT‐3′;

Itga9 forward primer: 5′‐ AAGTGTCGTGTCCATACCAAC‐3′,

Itga9 reverse primer: 5′‐ GGTCTGCTTCGTAGTAGATGTTC‐3′.

### Statistical Analysis

Data are presented as mean ± standard deviation. Differences between groups were tested by Student's t‐test or one‐way analysis of variance (ANOVA).

## Conflict of Interest

The authors declare no conflict of interest.

## Supporting information

Supporting Information

## Data Availability

The data that support the findings of this study are available from the corresponding author upon reasonable request.

## References

[1] a) Q. Chen , M. Chen , Z. Liu , Chem. Soc. Rev. 2019, 48, 5506; 31589233 10.1039/c9cs00271e

[2] M. S. Goldberg , Nat. Rev. Cancer 2019, 19, 587. 31492927 10.1038/s41568-019-0186-9

[3] T. J. Laskowski , A. Biederstadt , K. Rezvani , Nat. Rev. Cancer 2022, 22, 557. 35879429 10.1038/s41568-022-00491-0 PMC9309992

[4] J. D. Chan , J. Lai , C. Y. Slaney , A. Kallies , P. A. Beavis , P. K. Darcy , Nat. Rev. Immunol. 2021, 21, 769. 33879873 10.1038/s41577-021-00539-6

[5] A. D. Waldman , J. M. Fritz , M. J. Lenardo , Nat. Rev. Immunol. 2020, 20, 651. 32433532 10.1038/s41577-020-0306-5 PMC7238960

[6] D. J. Irvine , E. L. Dane , Nat. Rev. Immunol. 2020, 20, 321. 32005979 10.1038/s41577-019-0269-6 PMC7536618

[7] a) P. Sharma , J. P. Allison , Science 2015, 348, 56; 25838373 10.1126/science.aaa8172

[8] a) R. Kuai , W. M. Yuan , S. Son , J. Nam , Y. Xu , Y. C. Fan , A. Schwendeman , J. J. Moon , Sci. Adv. 2018, 4, eaao1736; 29675465 10.1126/sciadv.aao1736 PMC5906077

[9] a) C. L. Maynard , C. O. Elson , R. D. Hatton , C. T. Weaver , Nature 2012, 489, 231; 22972296 10.1038/nature11551 PMC4492337

[10] Y. Lee , N. Kamada , J. J. Moon , Adv. Drug Delivery Rev. 2021, 179, 114021. 10.1016/j.addr.2021.114021 PMC8665886 34710529

[11] Y. Zhang , M. Li , G. Du , X. Chen , X. Sun , Adv. Drug Delivery Rev. 2021, 177, 113928. 10.1016/j.addr.2021.113928 34411689

[12] L. A. O'Neill , E. J. Pearce , J. Exp. Med. 2016, 213, 15. 26694970 10.1084/jem.20151570 PMC4710204

[13] a) M. D. Buck , R. T. Sowell , S. M. Kaech , E. L. Pearce , Cell 2017, 169, 570; 28475890 10.1016/j.cell.2017.04.004 PMC5648021

[14] M. D. Buck , D. O'Sullivan , E. L. Pearce , J. Exp. Med. 2015, 212, 1345. 26261266 10.1084/jem.20151159 PMC4548052

[15] E. L. Mills , B. Kelly , A. Logan , A. S. H. Costa , M. Varma , C. E. Bryant , P. Tourlomousis , J. H. M. Dabritz , E. Gottlieb , I. Latorre , S. C. Corr , G. McManus , D. Ryan , H. T. Jacobs , M. Szibor , R. J. Xavier , T. Braun , C. Frezza , M. P. Murphy , L. A. O'Neill , Cell 2016, 167, 457. 27667687 10.1016/j.cell.2016.08.064 PMC5863951

[16] S. Willenborg , D. E. Sanin , A. Jais , X. Ding , T. Ulas , J. Nuchel , M. Popovic , T. MacVicar , T. Langer , J. L. Schultze , A. Gerbaulet , A. Roers , E. J. Pearce , J. C. Bruning , A. Trifunovic , S. A. Eming , Cell Metab. 2021, 33, 2398. 34715039 10.1016/j.cmet.2021.10.004

[17] a) D. Yang , Y. L. Zhao , H. Guo , Y. N. Li , P. Tewary , G. M. Xing , W. Hou , J. J. Oppenheim , N. Zhang , ACS Nano 2010, 4, 1178; 20121217 10.1021/nn901478z PMC2835518

[18] S. K. Biswas , A. Mantovani , Nat. Immunol. 2010, 11, 889. 20856220 10.1038/ni.1937

[19] a) J. K. Dowling , R. Afzal , L. J. Gearing , M. P. Cervantes‐Silva , S. Annett , G. M. Davis , C. De Santi , N. Assmann , K. Dettmer , D. J. Gough , G. R. Bantug , F. I. Hamid , F. K. Nally , C. P. Duffy , A. L. Gorman , A. M. Liddicoat , E. C. Lavelle , C. Hess , P. J. Oefner , D. K. Finlay , G. P. Davey , T. Robson , A. M. Curtis , P. J. Hertzog , B. R. G. Williams , C. E. McCoy , Nat. Commun. 2021, 12, 1460; 33674584 10.1038/s41467-021-21617-2 PMC7936006

[20] R. Fior , V. Povoa , R. V. Mendes , T. Carvalho , A. Gomes , N. Figueiredo , M. G. Ferreira , Proc. Natl. Acad. Sci. USA 2017, 114, E8234. 28835536 10.1073/pnas.1618389114 PMC5625889

[21] K. Honda , D. R. Littman , Nature 2016, 535, 75. 27383982 10.1038/nature18848

[22] a) C. J. Avraamides , B. Garmy‐Susini , J. A. Varner , Nat. Rev. Cancer 2008, 8, 604; 18497750 10.1038/nrc2353 PMC2577722

[23] M. Schieber , N. S. Chandel , Curr. Biol. 2014, 24, R453. 24845678 10.1016/j.cub.2014.03.034 PMC4055301

[24] A. M. Boutte , D. B. Friedman , M. Bogyo , Y. F. Min , L. Yang , P. C. Lin , FASEB J. 2011, 25, 2626. 21518852 10.1096/fj.10-180604 PMC3136340

[25] a) Y. Fujita , A. Khateb , Y. Li , R. Tinoco , T. Zhang , H. Bar‐Yoseph , M. A. Tam , Y. Chowers , E. Sabo , S. Gerassy‐Vainberg , E. Starosvetsky , B. James , K. Brown , S. S. Shen‐Orr , L. M. Bradley , P. A. Tessier , Z. A. Ronai , Cell Rep. 2018, 24, 3296; 30232010 10.1016/j.celrep.2018.08.057 PMC6185744

[26] a) R. Medzhitov , Nature 2007, 449, 819; 17943118 10.1038/nature06246

