# Supplementary material for: Oral Immunotherapy Reshapes Intestinal Immunosuppression via Metabolic Reprogramming to Enhance Systemic Anti‐Tumor Immunity
Source: Adv Sci (Weinh). 2023 Oct 26;10(35):2302910. doi: 10.1002/advs.202302910 (PMC10724426; doi:10.1002/advs.202302910)
Supplement: Supplementary file 1 — Supporting Information [file ADVS-10-2302910-s001.pdf]

ADVANCED SCIENCE
Open Access

Supporting Information

for *Adv. Sci.*, DOI 10.1002/advs.202302910

Oral Immunotherapy Reshapes Intestinal Immunosuppression via Metabolic Reprogramming to Enhance Systemic Anti-Tumor Immunity

Xinran Cao, Yuan Xu, Chen Zhou, Jiawei Huo, Shenge Su, Lei Liu, Ziran Zhu, Lei Li, Wang Jia, Chunru Wang and Mingming Zhen**

Supporting Information

Oral immunotherapy reshapes intestinal immunosuppression via metabolic reprogramming to enhance systemic anti-tumor immunity

Xinran Cao†, Yuan Xu†, Chen Zhou, Jiawei Huo, Shenge Su, Lei Liu, Ziran Zhu, Lei Li, Wang Jia, Chunru Wang* and Mingming Zhen*

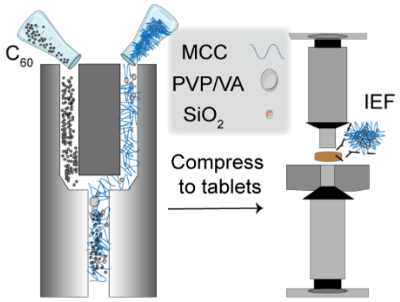

Fig. S1. Schematic illustration of IEF preparation.

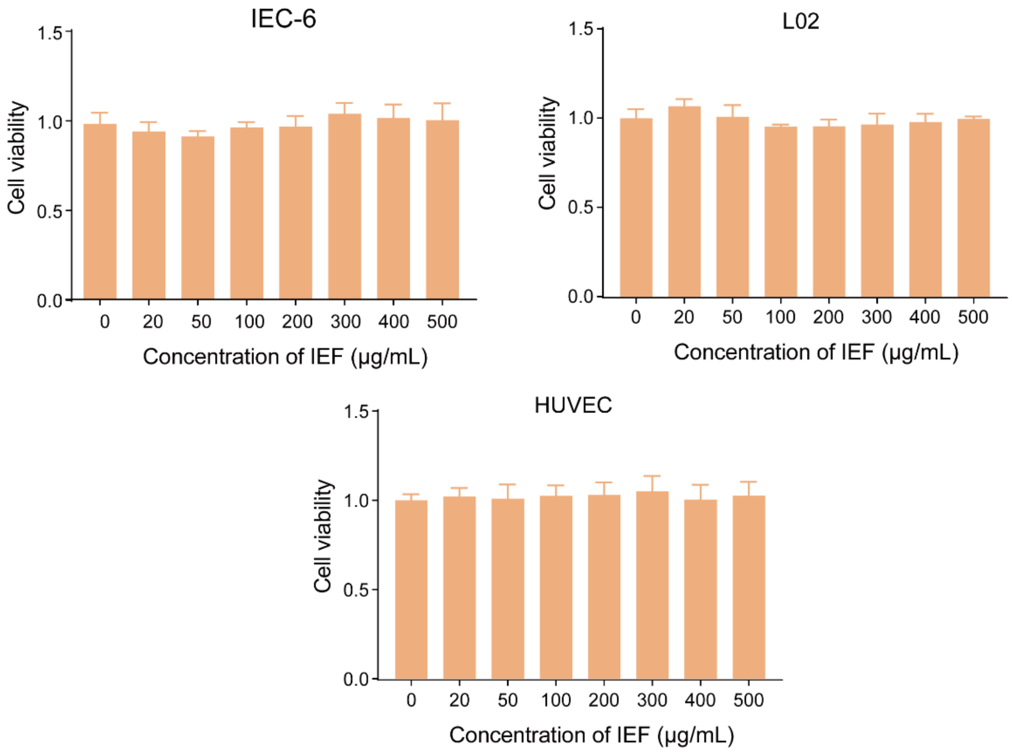

Fig. S2. Cellular viabilities of IEC-6, L02, and HUVEC after co-incubation with different concentrations of IEF (n=6).

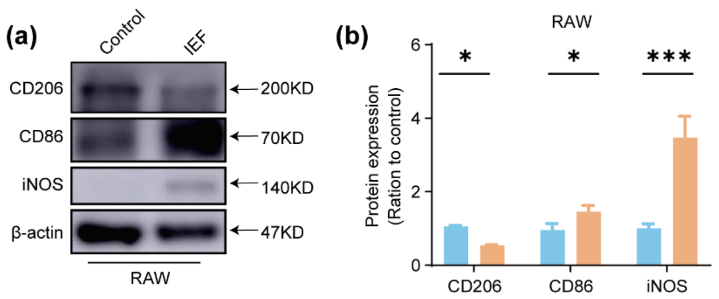

Fig. S3. Polarization ability of IEF on RAW macrophages. a-b) The protein expressions of M2 marker (CD206), and M1 markers (CD86, iNOS) in RAW macrophages before and after co-incubation with IEF (n=3). The data are shown as the mean ± s.d. Student's t-test, *p < 0.05, **p < 0.01, ***p < 0.001.

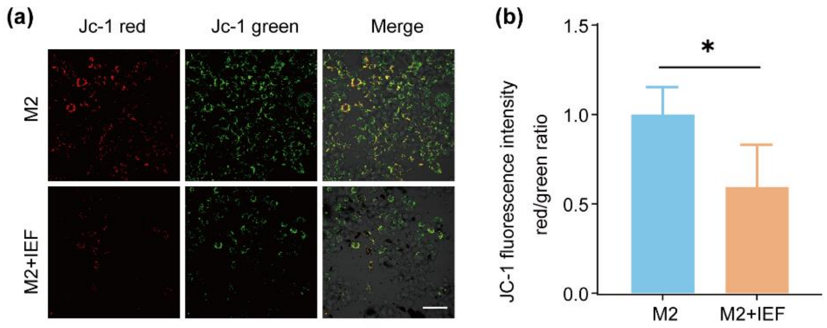

Fig. S4. Effect of IEF on mitochondrial membrane potential in M2 macrophages. a-b) Representative fluorescence images in mitochondrial membrane potential (JC-1) before and after co-incubation with IEF of M2 macrophages, (**b**) and quantification (n=4). Scale bar, 40 μm.

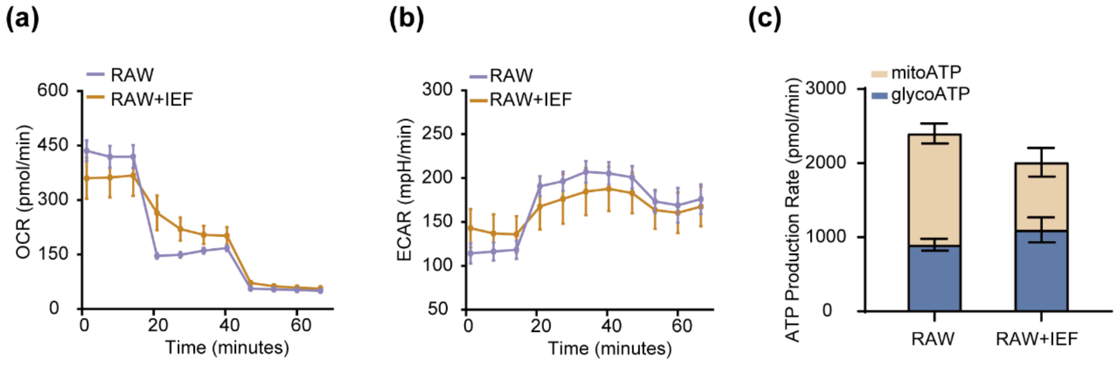

Fig. S5. Analysis of energy metabolism in RAW macrophages treated with IEF. a-c) The measurement flow and quantitative analysis of mitochondrial ATP (mitoATP)

production rate and glycolytic ATP (glyATP) production rate of M0-type macrophages after IEF treatment (n=6). The data are shown as the mean ± s.d. Student's t-test, *p < 0.05, **p < 0.01, ***p < 0.001.

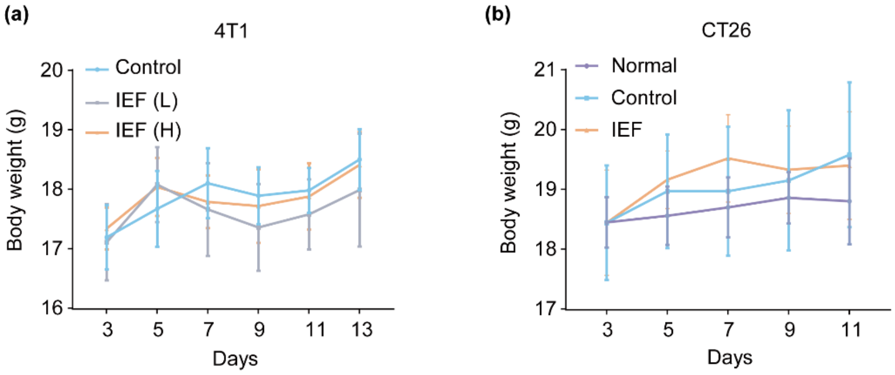

Fig. S6. Changes in body weight of different mice before and after IEF treatment (n=8).

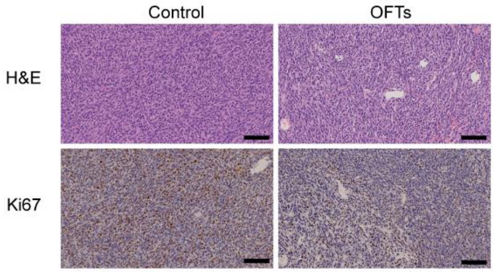

Fig. S7. Representative images of H&E, and Ki67 staining sections of tumor from different treatment groups. Images were representative of four biologically independent mice in each group. Scale bar, 100 µm.

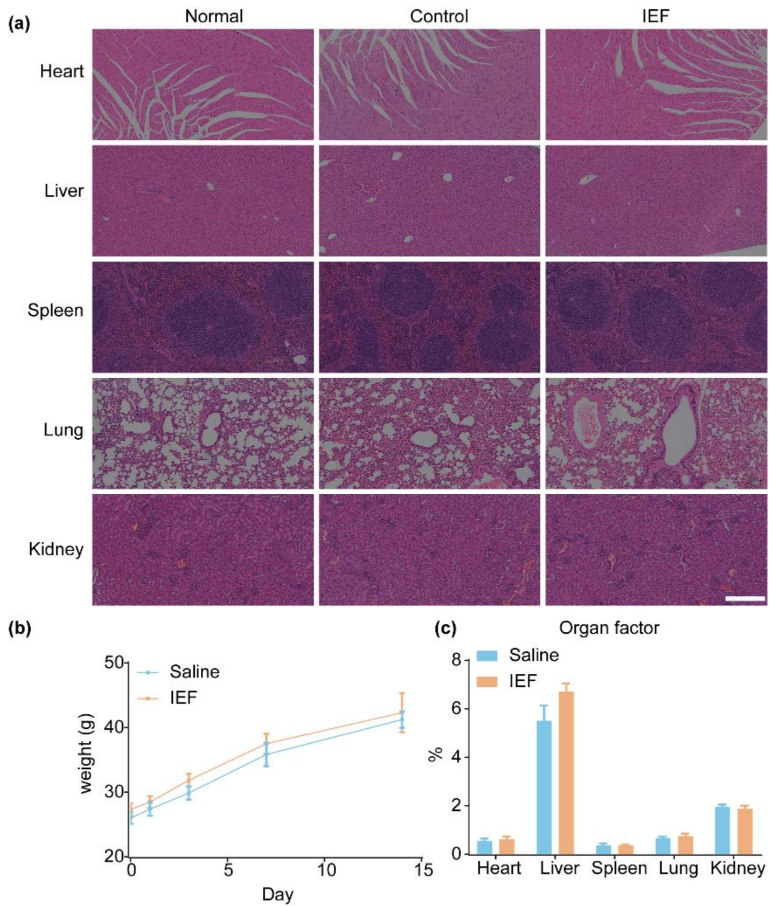

Fig. S8. The bio-safety of IEF. a) Representative images of H&E staining slices of heart, liver, spleen, lung, and kidney at the end of treatment in mice with CT26 cells. Scale bar, 250 μm. **b)** Weight growth curve of KM mouse acute toxicity experiment. **c)** Organ coefficient of KM mouse acute toxicity experiment (n=10).

	Liver	Spleen	Lung	Kidney	Blood	Tumor	Urine
1 h	ND±ND	ND±ND	ND±ND	ND±ND	ND±ND	ND±ND	
4 h	ND±ND	ND±ND	ND±ND	ND±ND	ND±ND	ND±ND	
8 h	ND±ND	ND±ND	ND±ND	ND±ND	ND±ND	ND±ND	
12h	ND±ND	ND±ND	ND±ND	ND±ND	ND±ND	ND±ND	ND±ND

Table S1. The distribution of IEF in mice at different time points. (n=6; ND represents no detection; The minimum detection limit is 50 ng/g.)

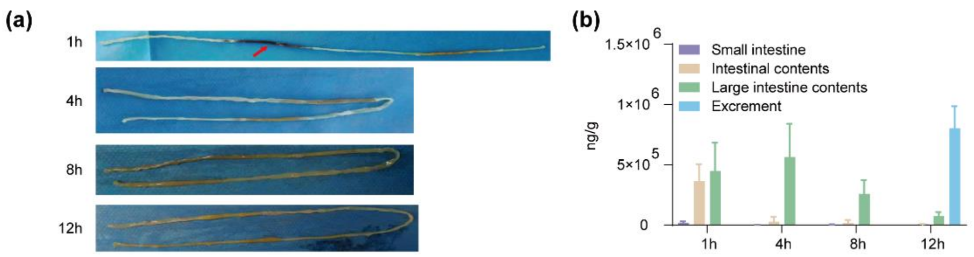

Fig. S9. The biodistribution of IEF. a) Distribution of IEF in the small intestine at different time points. Red arrow, IEF. **b)** Quantitative distribution of IEF in different parts and contents of the intestine at different time points (n=6).

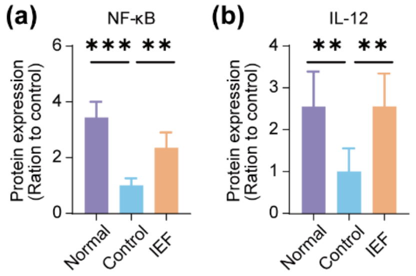

Fig. S10. The protein expression of NF-κB and IL-12 in the ileum was measured by ELISA (n=5). The data are shown as the mean ± s.d. One-way ANOVA, *p < 0.05, **p < 0.01, ***p < 0.001.

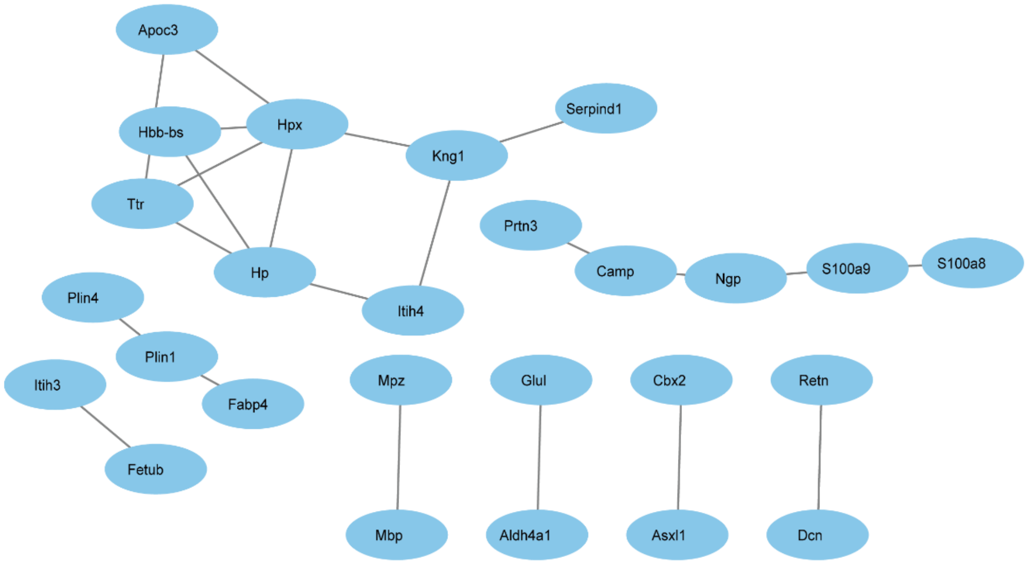

Fig. S11. Tumor site proteomics analysis. Protein interaction network between differential expression proteins in tumors**.**

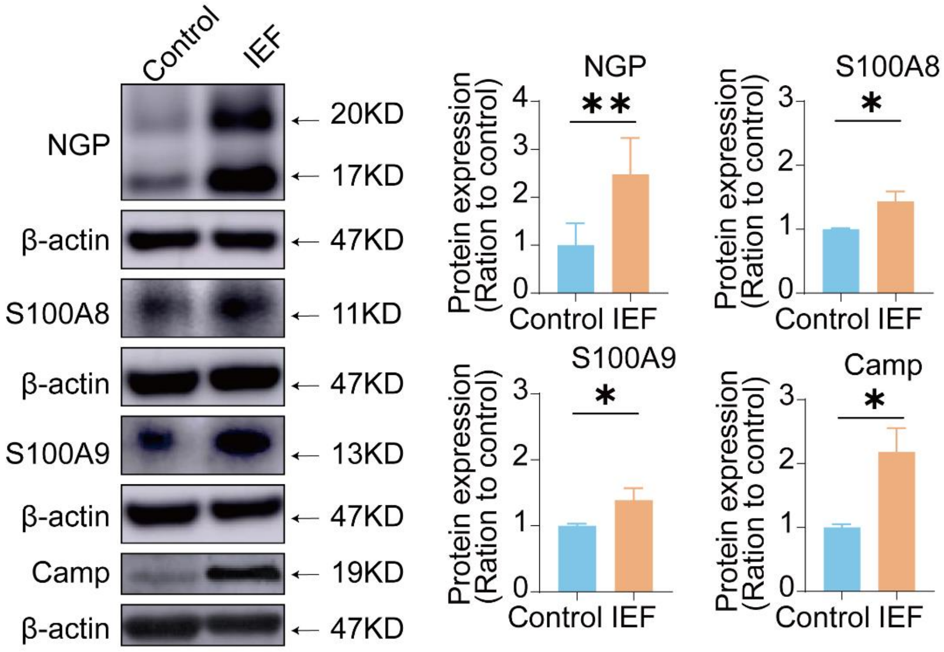

Fig. S12. Detection of immune-related proteins at tumor sites. WB assay and quantitative protein expressions of NGP, S100A8, S100A9 and CAMP (n=3)**.** The data are shown as the mean ± s.d. Student's t-test, *p < 0.05, **p < 0.01, ***p < 0.001.

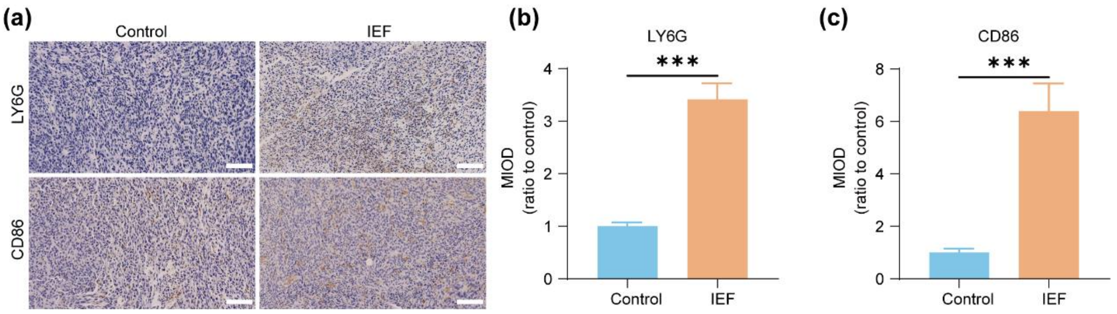

Fig. S13. Intrinsic immune response at the tumor site. a) Immunohistochemical staining of neutrophil (labeled by LY6G) and M1-type macrophages (labeled by CD86) in tumor. Scale bar, 100 µm. **b)** Quantification of neutrophils expression (LY6G) in the tumor. **c)** Quantification of M1 macrophage expression (CD86) in the tumor (n=5). The data are shown as the mean ± s.d. Student's t-test, *p < 0.05, **p < 0.01, ***p < 0.001.
